# Infiltrated plaques on the lateral aspects of the hips disclosing a case of adult gluteal granuloma^☆^^☆☆^

**DOI:** 10.1016/j.abd.2020.06.029

**Published:** 2021-07-15

**Authors:** Anna Karoline Gouveia de Oliveira, Neusa Yuriko Sakai Valente, Thais do Amaral Carneiro Cunha, Agatha Ramos Oppenheimer

**Affiliations:** Hospital do Servidor Público Estadual, São Paulo, SP, Brazil

Dear Editor,

Adult gluteal granuloma (AGG) is an inflammatory dermatosis that occurs in adults or the elderly on skin exposed to prolonged contact with feces and urine. It presents as well-demarcated areas of erythema, edema, scaling, papules, nodules, erosions, and ulcerations. On histopathological analysis, there is no granuloma, but there is acanthosis, spongiosis, varying degrees of a mixed superficial and deep inflammatory infiltrate, and proliferation of vessels in the dermis.^1^ It is a rare condition, with few reports in the literature and it is classically known to occur in children.

An 86-year-old man presented with erythematous, infiltrated, well-defined plaques, with a slightly scaling border and central clearing, asymptomatic, on the lateral aspects of the hips for six months (Figure 1). The patient had a history of diabetes mellitus, radical prostatectomy due to adenocarcinoma, urinary incontinence and recurrent urinary infections. Complementary tests showed glycated hemoglobin of 10.3% and urinary pH of 8.5. Direct mycological examination of the lesion was negative and the histopathological analysis showed acanthosis, spongiosis mainly in the acrosyringial ducts, a discrete perivascular dermal inflammatory infiltrate with lymphocytes, histiocytes, eosinophils, plasmocytes, and red blood cell extravasation (Figure 2). The Grocott stain was negative for fungi. After behavioral measures to keep the diaper area dry and 0.05% fluticasone propionate cream, the lesions disappeared.

**Figure 1:**
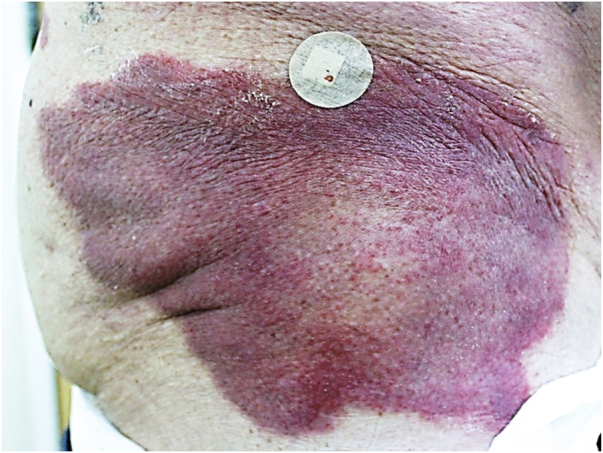
Erythematous infiltrated, well-delimited plaque, with a slightly scaling edge on the right side of the hip.

**Figure 2:**
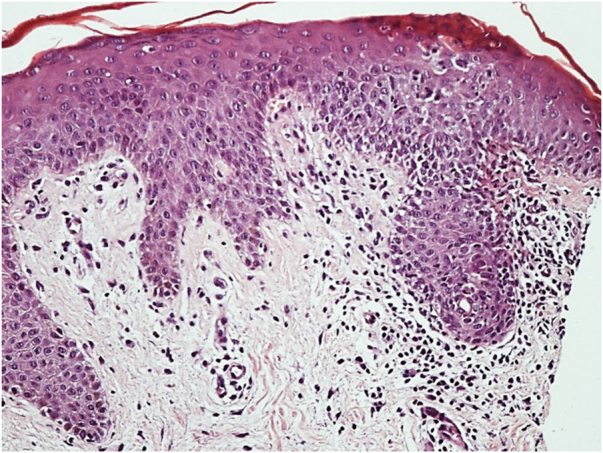
Histopathology: Epidermis with acanthosis and spongiosis mainly affecting the acrosyringial ducts and a mild superficial perivascular inflammatory infiltrate in the dermis (Hematoxylin & eosin, ×200).

Gluteal granuloma is an inflammatory condition, most commonly observed in infanthood, in the form of papules and nodules in the diaper area. Of uncertain etiology, it is believed that a skin reaction occurs to the repeated contact with irritating substances present in feces, urine and cleaning utensils. Infection by *Candida albicans*, occlusion and halogenated corticosteroids are possible pathogenic factors.^2^

The pH of normal skin is approximately 5.5; while that of urine is around 6 or higher, when there is bacteriuria or infection by urease-producing bacteria, an environment that increases the activity of lipases and proteases, impairs the skin barrier and predisposes to the action of local irritants. Our patient had a urinary pH of 8.5 and bacteriuria, corroborating the findings reported by Isogai et al.^3^

The distribution of the lesions is related to the patient’s position, with nodules located in the scrotum and labia majora of patients who remain seated for a long time, and nodules around the anus in bedridden patients.^3^ Our patient had the diapers frequently changed during the day and, when sleeping, remained in lateral decubitus, associated with polyuria due to decompensated diabetes, factors that caused the lesions on the lateral aspects of the hips.

Recently, dermoscopic findings have been described in a case of AGG, showing papillae with rounded white areas surrounded by erythema and serrated white borders. These papillae were separated by fissures and contained a whitish mesh, comedo-like openings and dotted vessels under the greatest magnification.^4^

Keeping the skin clean and dry is the main treatment and the best form of prevention. There are individual reports or case series and, in most of them, topical treatments, including corticosteroids, have failed. The use of topical corticosteroids was effective in two patients.^5^

With the aging of the population and the increase in the number of incontinent individuals, we will possibly face this entity more frequently, justifying the importance of learning about it.

## Financial support

None declared.

## Authors’ contributions

Anna Karoline Gouveia de Oliveira: Drafting and editing of the manuscript.

Neusa Yuriko Sakai Valente: Approval of the final version of the manuscript; drafting and editing of the manuscript; intellectual participation in propaedeutic and/or therapeutic conduct of the studied cases; critical review of the manuscript.

Thais do Amaral Carneiro Cunha: Intellectual participation in propaedeutic and/or therapeutic conduct of the studied cases.

Agatha Ramos Oppenheimer: Intellectual participation in propaedeutic and/or therapeutic conduct of the studied cases.

## Conflicts of interest

None declared.
